# Supplementary figures and images for: Efficient population coding depends on stimulus convergence and source of noise
Source: PLoS Comput Biol. 2021 Apr 26;17(4):e1008897. doi: 10.1371/journal.pcbi.1008897 (PMC8075262; doi:10.1371/journal.pcbi.1008897)

**A**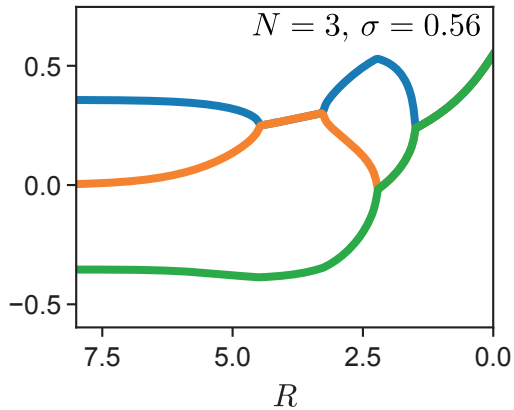**B**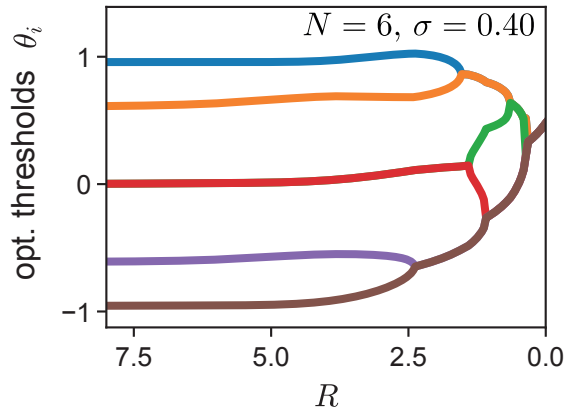

Supplement: S1 Fig — A. For N = 3 neurons there is a non-monotonicity of the number of distinct optimal thresholds with output noise for a relatively small input noise parameter range (0.54 < σ < 0.6, see Fig 4A). For high output noise (low R), first the upper two thresholds merge, before they split again with decreasing output noise and for even lower output noise the middle threshold merges with the lower threshold. B. For N = 6 neurons a similar transformation of thresholds happens, where the two middle thresholds split with decreasing output noise, thus increasing number of distinct optimal thresholds. (PDF) [file pcbi.1008897.s001.pdf]

**A** $N=4$ 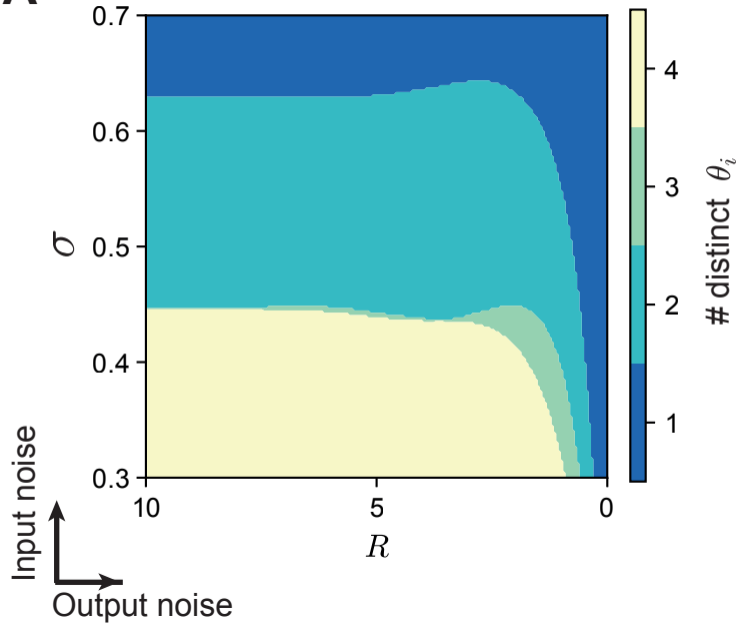**B** $N=6$ 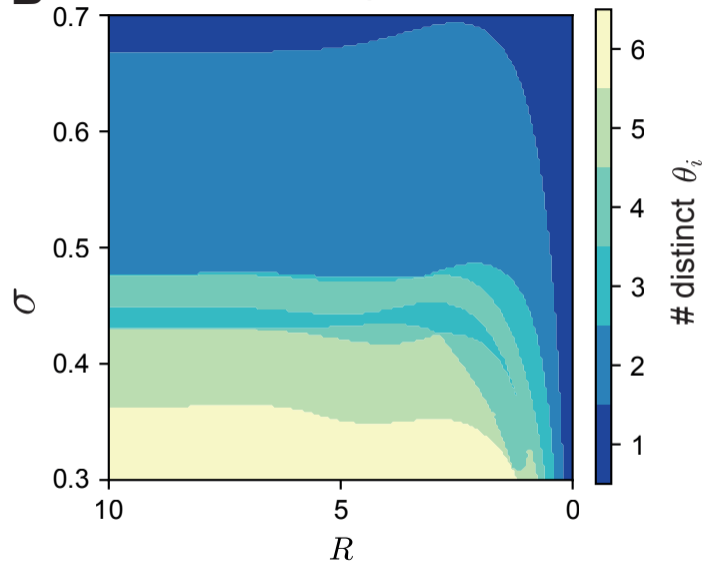

Supplement: S2 Fig — A. Number of distinct optimal thresholds for N = 4 cells depending on input noise σ and output noise R. B. As in A but for N = 6. (PDF) [file pcbi.1008897.s002.pdf]

**A**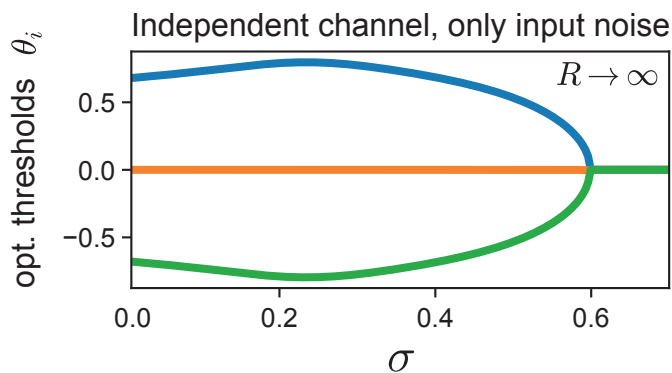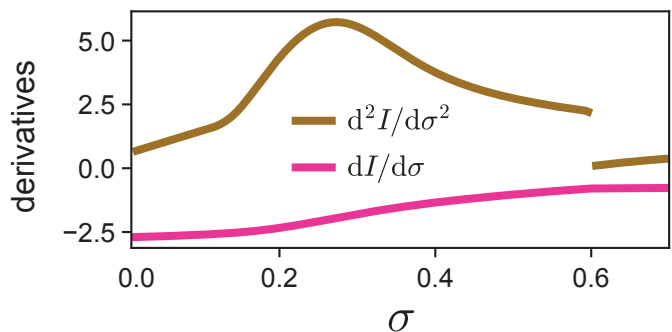**B**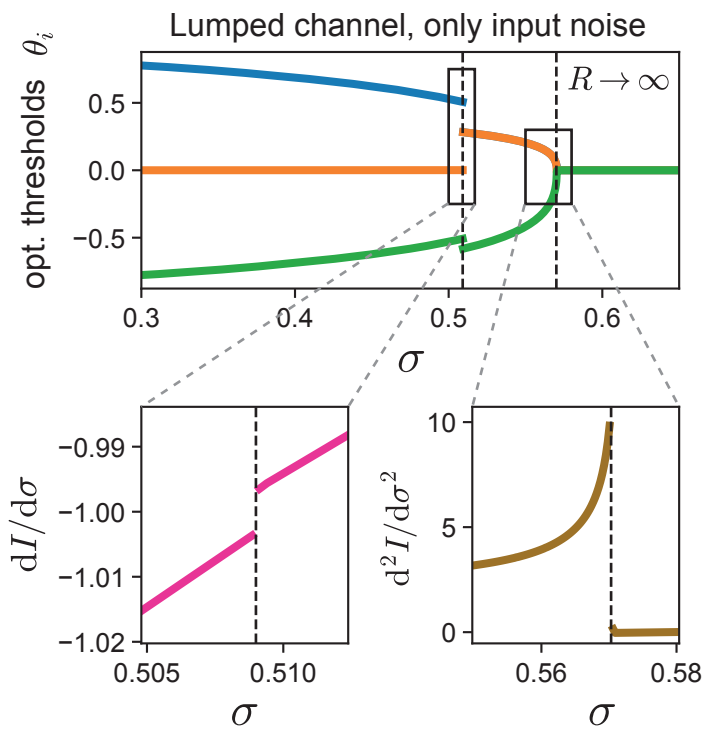**C**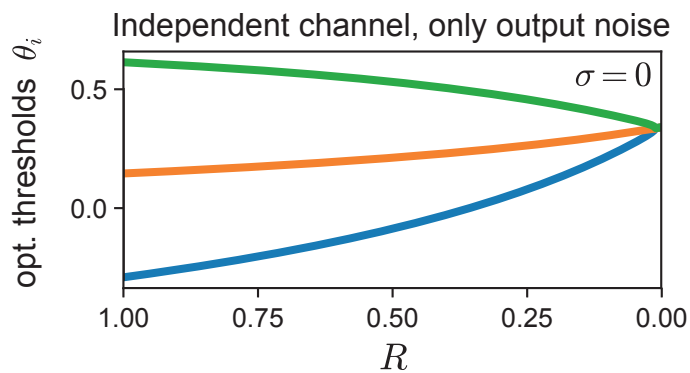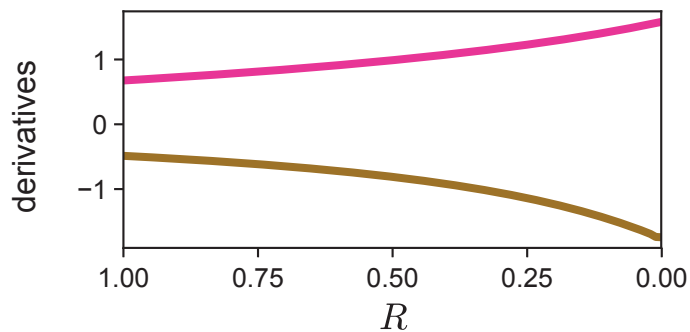**D**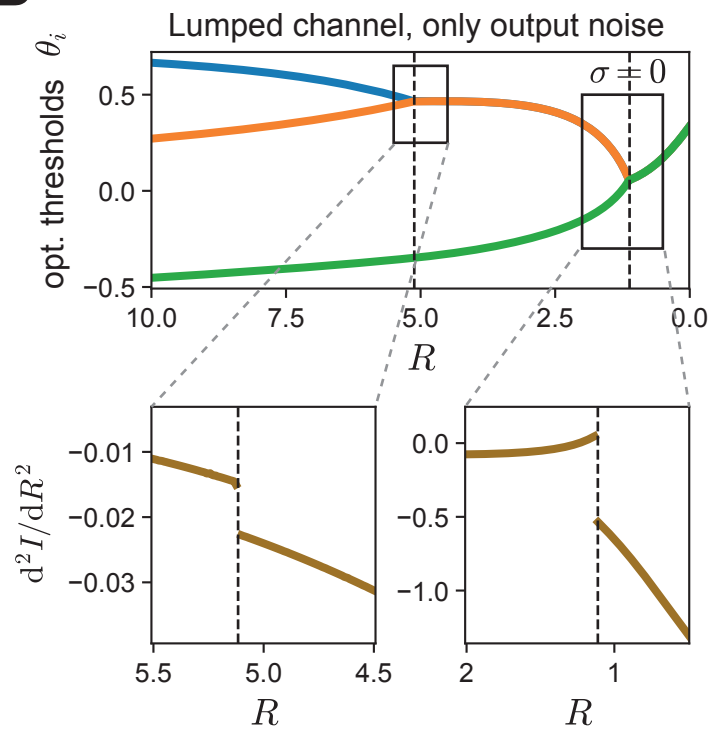

Supplement: S3 Fig — A. Threshold bifurcations for the independent-coding channel with respect to input noise σ for vanishing output noise. The derivatives of mutual information with respect to input noise indicate a second-order phase transition. B. As in A but for the lumped-coding channel. There is a first-order phase transition for low noise (left inset) and a second-order phase transition for high noise (right inset). C. The Independent-coding channel with vanishing input noise. No phase transition is visible since the “bifurcation” happens in the limit of infinite output noise. D. The Lumped-coding channel with vanishing input noise exhibits second-order phase transitions. (PDF) [file pcbi.1008897.s003.pdf]

**A**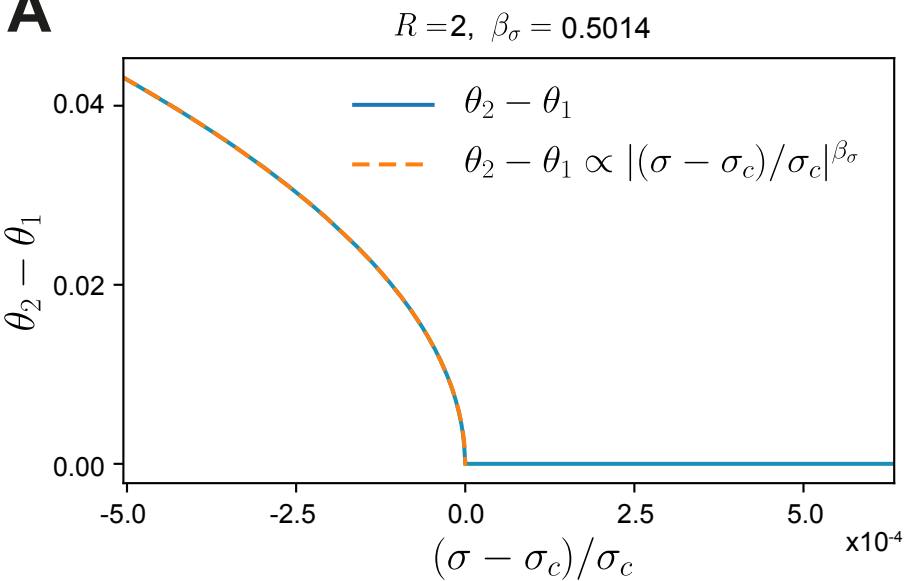**B**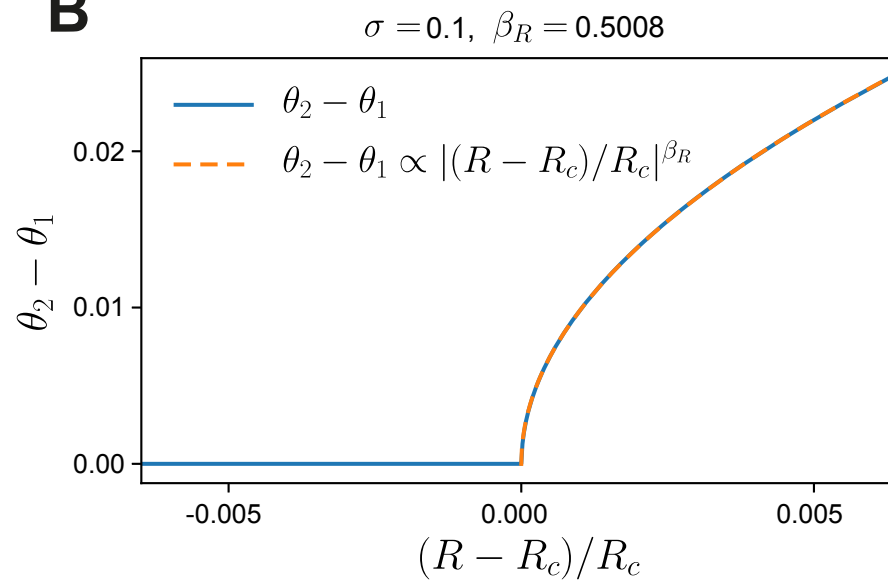**C**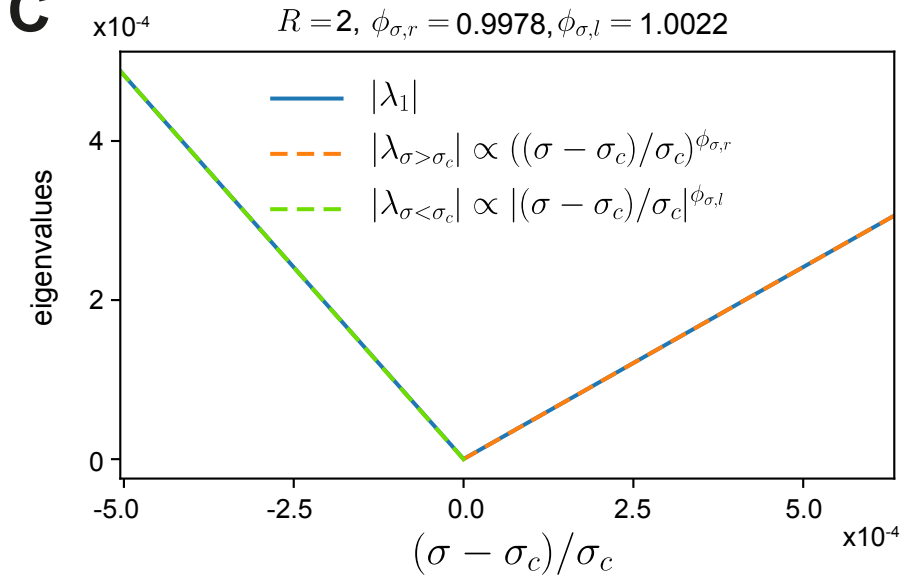**D**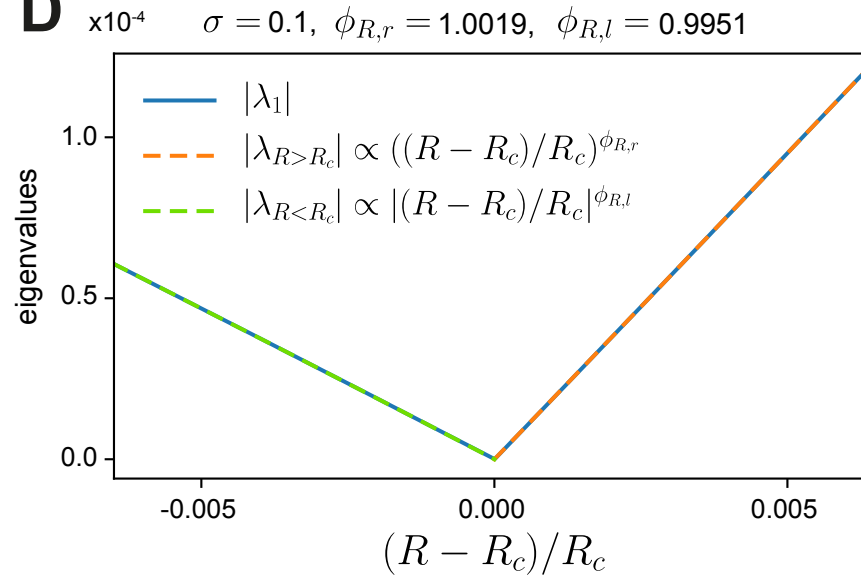

Supplement: S4 Fig — A. Obtaining the critical exponent βσ by fitting a monomial function to the threshold differences for input noise values slightly smaller than the critical input noise value σc. B. As in A, the critical exponent βR is obtained by fitting output noise values slightly smaller than the critical output noise Rc. C,D. Similarly, one obtains the critical exponents of the eigenvalues of the Hessian matrix of the information landscape, ϕl and ϕr, by fitting the eigenvalues for both slightly smaller (ϕl) and slightly larger (ϕr) noise values than the critical noise value. (PDF) [file pcbi.1008897.s004.pdf]

**A**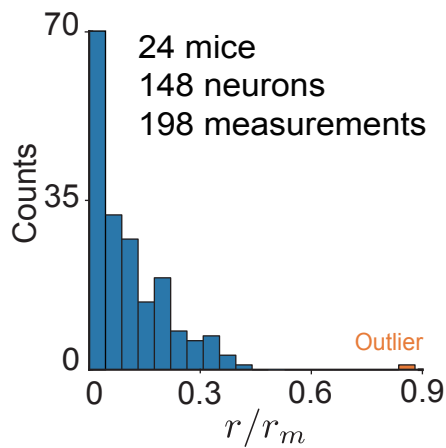**B**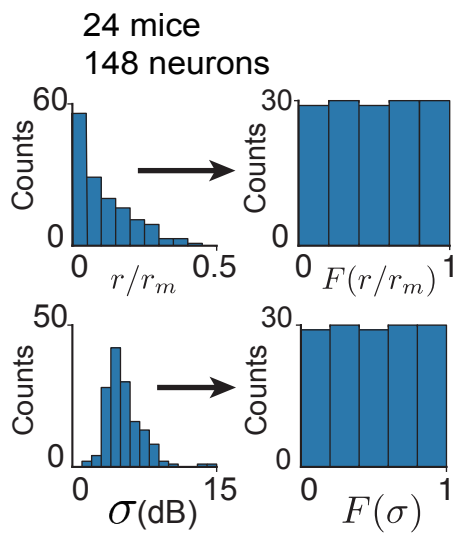**C**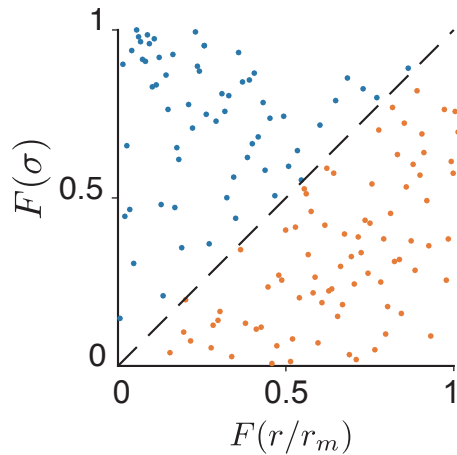**D**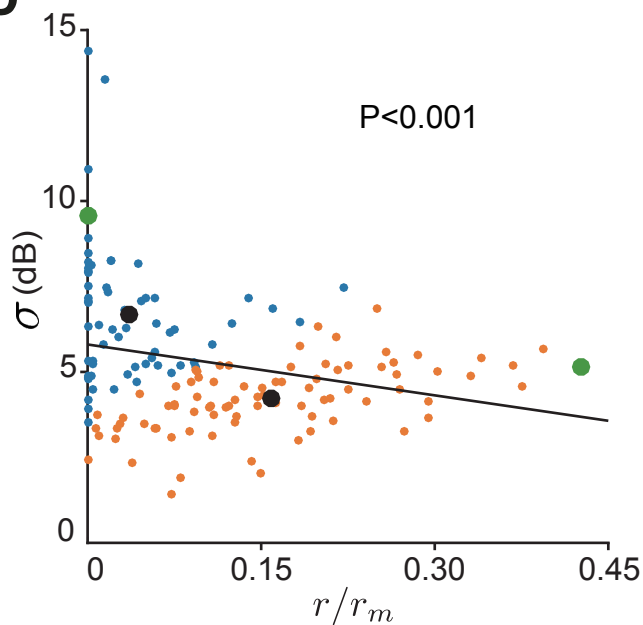**E**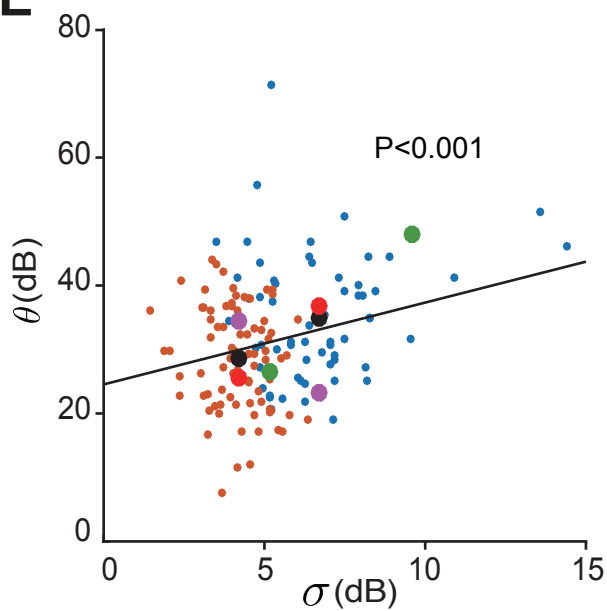

Supplement: S5 Fig — A. Distribution of normalized spontaneous firing rate (SR, r/rm). An outlier is identified and marked in orange. B. Transforming normalized SR r/rm and dynamic range σ into cumulative distribution functions. C. A diagram showing the method to classify neurons into two types. D. Scatter plot and linear fit between normalized SR r/rm and dynamic range σ. Black dots denote the ‘center of mass’ within each ‘type’, and the green dots show the values of example neurons in Fig 7B. E. As in D but for the relationship between σ and threshold θ. Magenta dots and red dots denote where mutual information is maximized, with corresponding σ1 and σ2 as the black dots. Average firing rate corresponding to red dots are lower. (PDF) [file pcbi.1008897.s005.pdf]

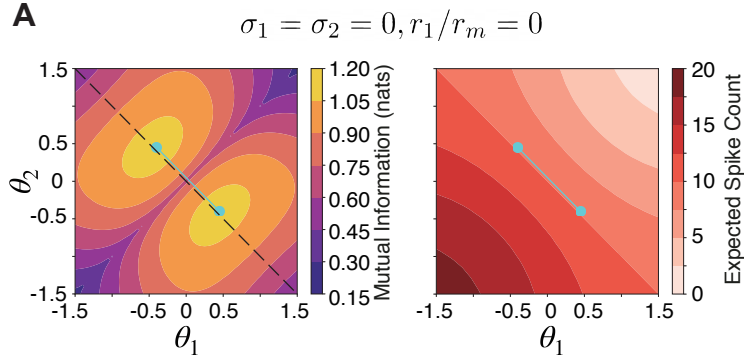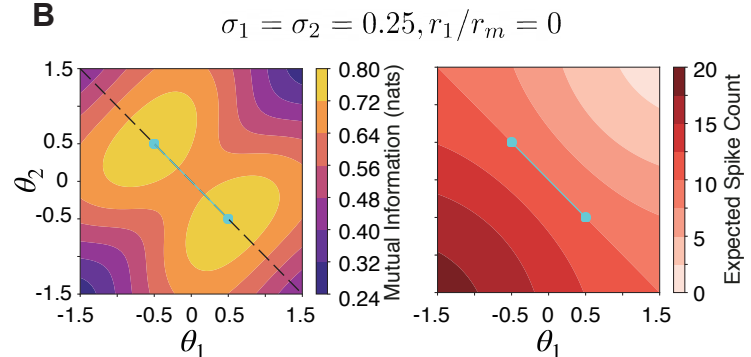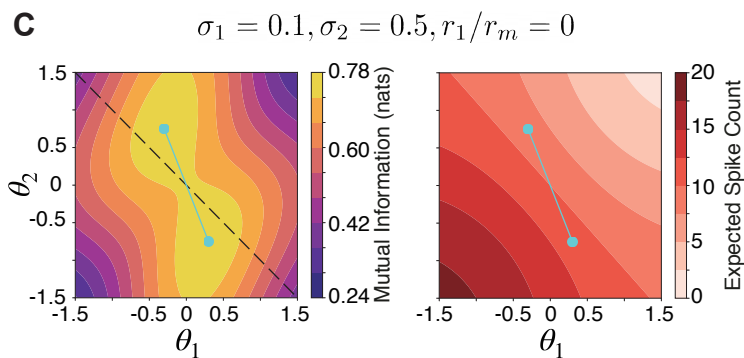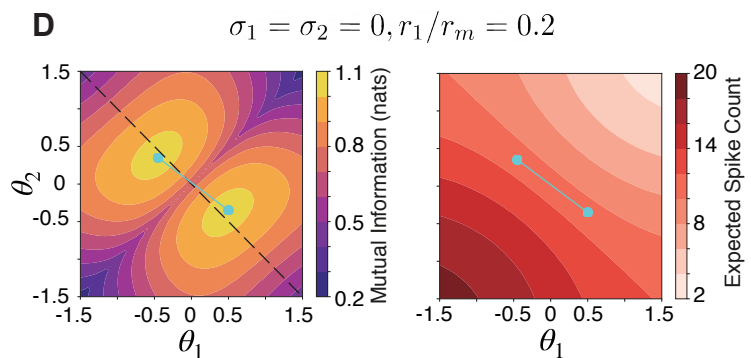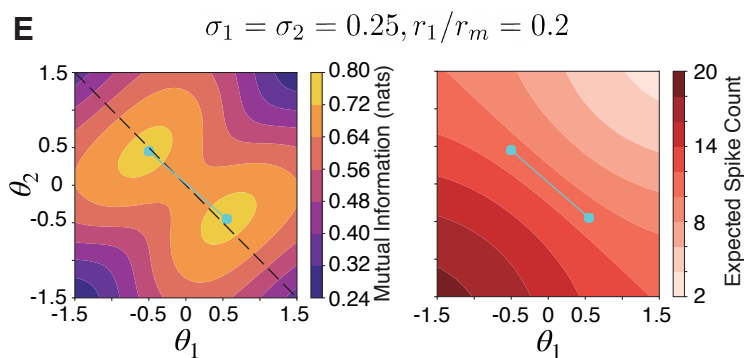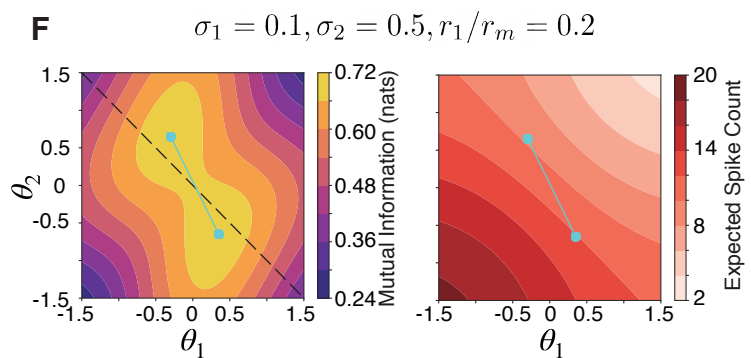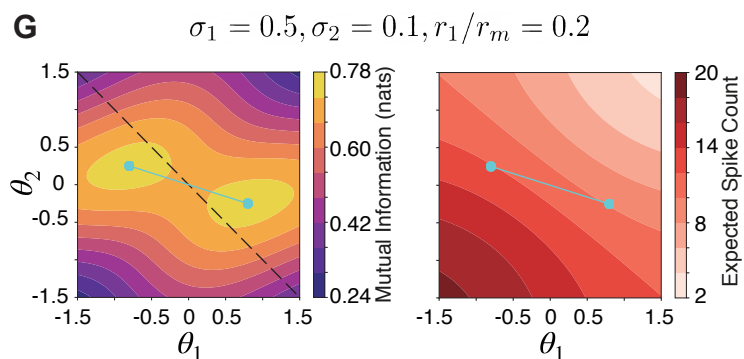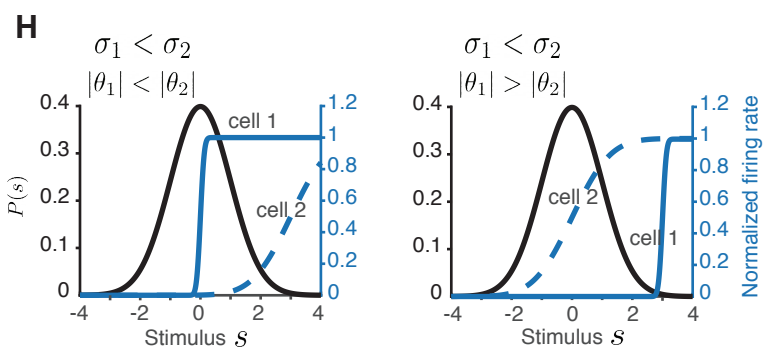

Supplement: S6 Fig — In each panel, the left plot corresponds to mutual information and the right one shows average firing rate. Cyan dots show optimal thresholds (θ1, θ2) which maximize mutual information. Maximal spike count is set to R = 10 for every panel. A. Both neurons have zero input noise σ1 = σ2 = 0, and zero spontaneous rate r1/rm = 0. B. The two neurons have identical but nonzero input noise σ1 = σ2 = 0.25, and zero spontaneous rate r1/rm = 0. C. The two neurons have two different and nonzero input noise σ1 = 0.1, σ2 = 0.5, and zero spontaneous rate r1/rm = 0. D. Both neurons have zero input noise σ1 = σ2 = 0, and nonzero spontaneous rate r1/rm = 0.2. E. The two neurons have identical but nonzero input noise σ1 = σ2 = 0.25, and non-zero spontaneous rate r1/rm = 0.2. F. The two neurons have two different and nonzero input noise σ1 = 0.1, σ2 = 0.5, and nonzero spontaneous rate r1/rm = 0.2. G. σ1 = 0.5, σ2 = 0.1, r1/rm = 0.2. H. The mechanism behind symmetry breaking of the mutual information landscape. The case (left) where the neuron with the larger input noise has a larger threshold located in the region where the stimulus rarely occurs is more efficient than in the case (right) where the neuron with the larger input noise has a smaller threshold near the stimulus mean where its dynamic range covers a large range of possible stimuli. (PDF) [file pcbi.1008897.s006.pdf]

**A**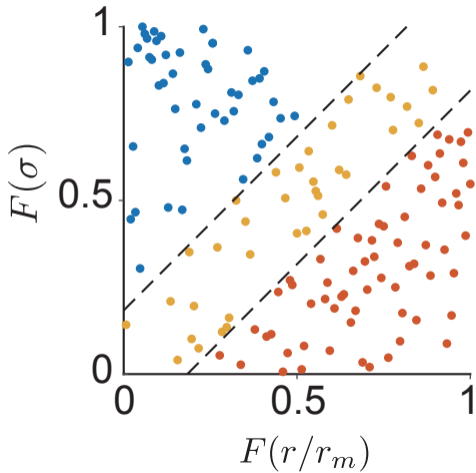**B**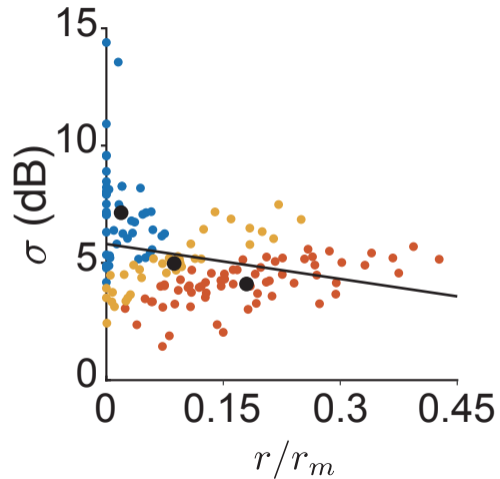**C**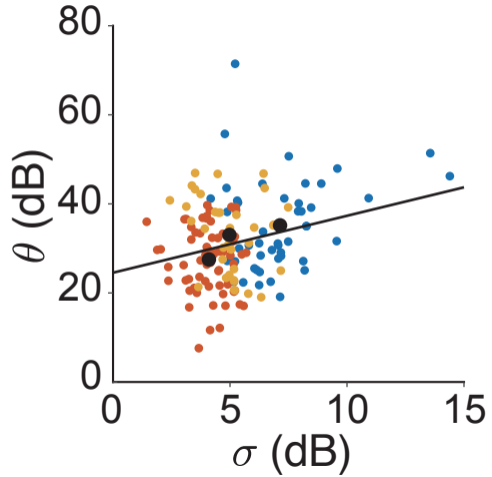

Supplement: S7 Fig — A. A diagram showing the method to classify neurons into three types. B. Scatter plot and linear fit between normalized spontaneous firing rate (SR, r/rm) and dynamic range σ. Black dots denote the ‘center of mass’ within each ‘type’. C. As in B but for the relationship between σ and threshold θ. (PDF) [file pcbi.1008897.s007.pdf]

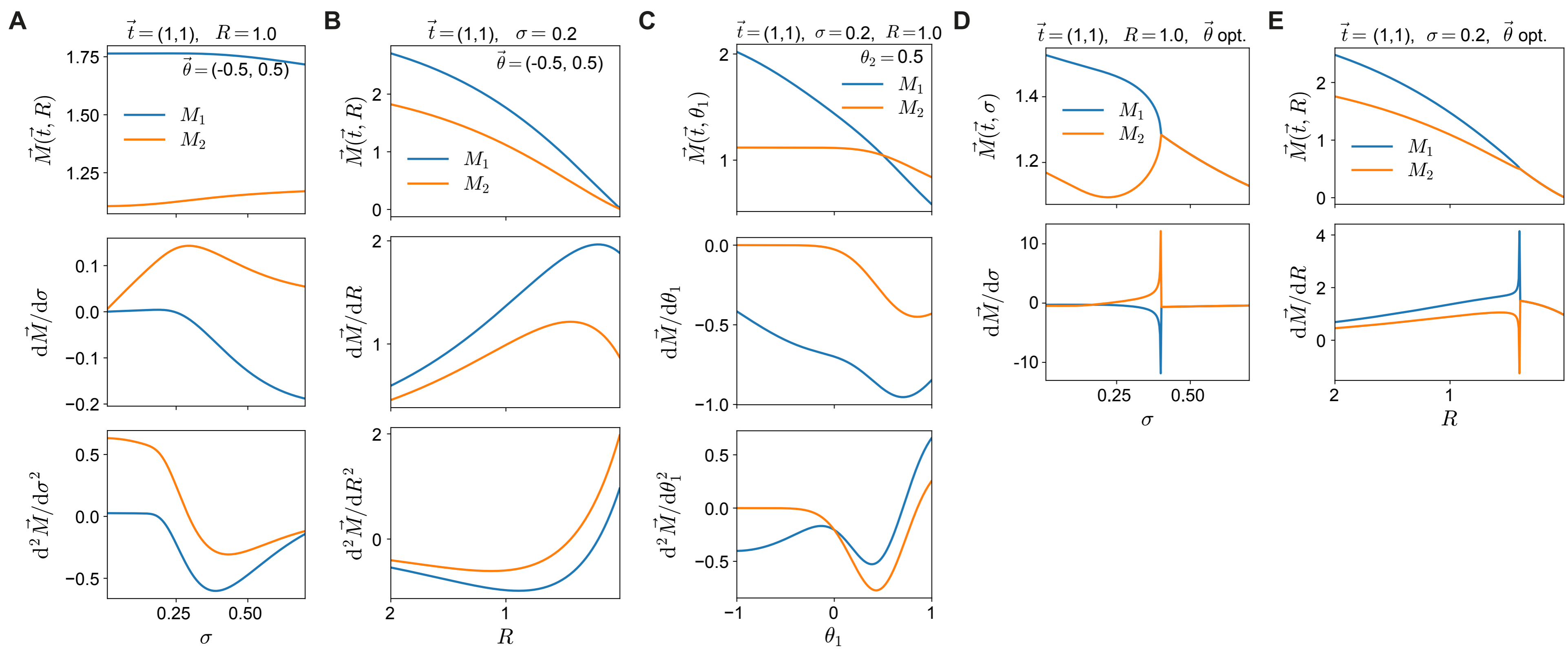

Supplement: S8 Fig — A. The two components (N = 2) of the moment-generating function M→(t→) for t→=(1,1) (see S1 Text) depending on input noise σ. Output noise value and threshold vector are fixed to R = 1 and θ→=(-0.5,0.5), respectively. The first two derivatives show no discontinuities. B. As A but depending on R with σ = 0.2. C. As A,B but depending on first threshold vector θ1. D,E. As A,B but with optimized threshold vector for each noise value. The components of the moment-generating function show a bifurcation and the first derivatives show discontinuities. (PDF) [file pcbi.1008897.s008.pdf]
