# Supplementary material for: Efficient population coding depends on stimulus convergence and source of noise
Source: PLoS Comput Biol. 2021 Apr 26;17(4):e1008897. doi: 10.1371/journal.pcbi.1008897 (PMC8075262; doi:10.1371/journal.pcbi.1008897)
Supplement: S9 Fig — Instead of a Gaussian stimulus and noise distribution we also used a generalized normal distribution and varied the kurtosis (small β means high kurtosis, see Eq 25). A. Laplacian (having high kurtosis) as input distribution. B. Input distribution with low kurtosis (similar to uniform). C. Laplace distribution as noise distribution. D. Noise distribution with low kurtosis. (PDF) [file pcbi.1008897.s009.pdf]

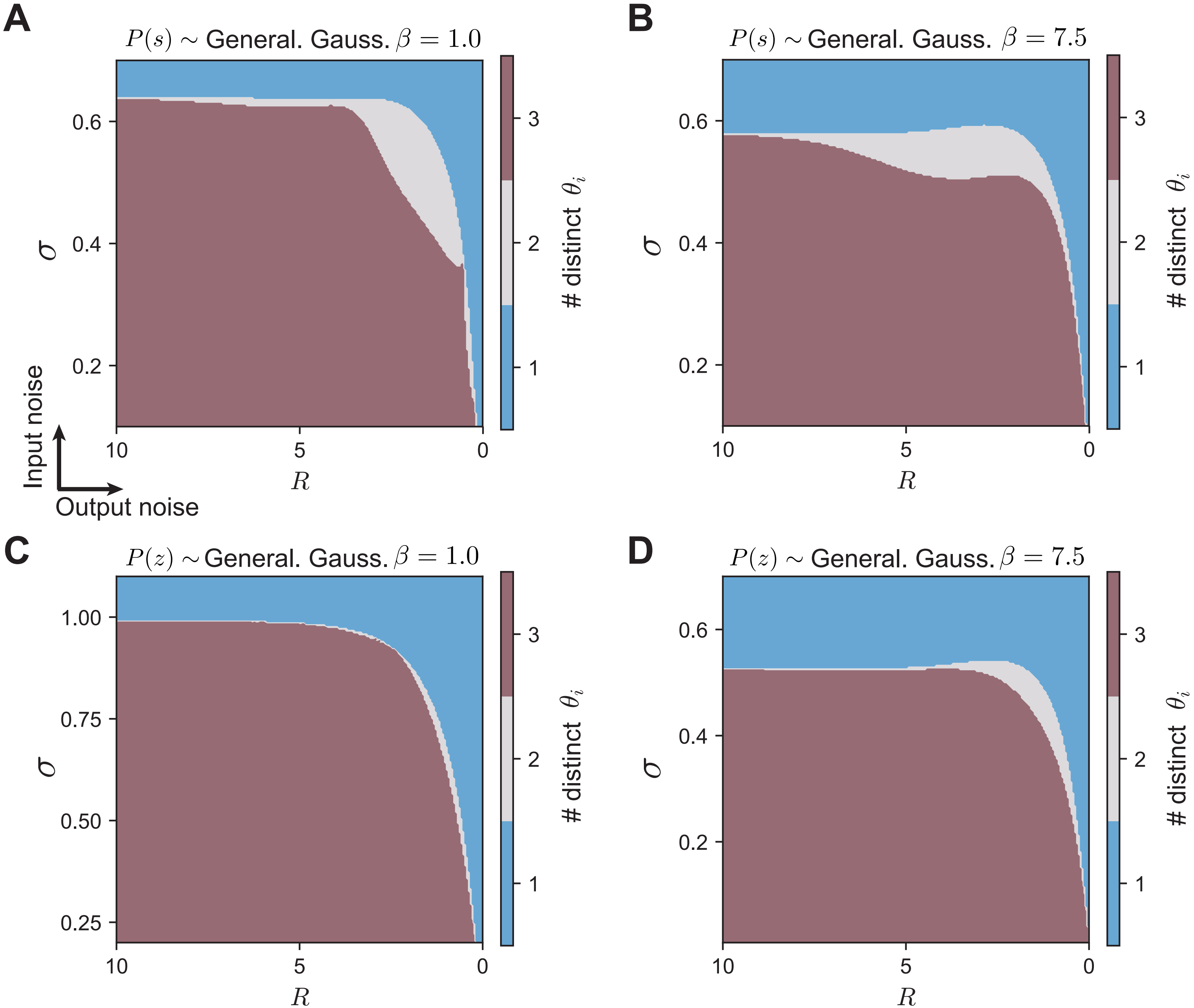

A
$P(s) \sim$ General. Gauss. $\beta = 1.0$
σ
0.6
0.4
0.2
10
5
0
R
distinct θ_i
3
2
1
Input noise
Output noise
B
$P(s) \sim$ General. Gauss. $\beta = 7.5$
σ
0.6
0.4
0.2
10
5
0
R
distinct θ_i
3
2
1
C
$P(z) \sim$ General. Gauss. $\beta = 1.0$
σ
1.00
0.75
0.50
0.25
10
5
0
R
distinct θ_i
3
2
1
D
$P(z) \sim$ General. Gauss. $\beta = 7.5$
σ
0.6
0.4
0.2
10
5
0
R
distinct θ_i
3
2
1
