# Supplementary material for: Efficient population coding depends on stimulus convergence and source of noise
Source: PLoS Comput Biol. 2021 Apr 26;17(4):e1008897. doi: 10.1371/journal.pcbi.1008897 (PMC8075262; doi:10.1371/journal.pcbi.1008897)
Supplement: S1 Text — (PDF) [file pcbi.1008897.s010.pdf]

S1 Text

Efficient population coding depends on stimulus convergence and source of noise

Kai Röth[1,2], Shuai Shao[1,3], Julijana Gjorgjieva[1,2,*]

1 Computation in Neural Circuits Group, Max Planck Institute for Brain Research, Frankfurt, Germany
2 School of Life Sciences, Technical University of Munich, Freising, Germany
3 Donders Institute and Faculty of Science, Radboud University, Nijmegen, Netherlands

* gjorgjieva@brain.mpg.de

The moment-generation function of the independent-coding channel

Following [1], the moment-generating function of the N-dimensional random variable $\vec{X}$ is:

$$M_{\vec{X}}(\vec{t}) = \langle e^{\vec{t}\cdot\vec{X}} \rangle, \qquad \vec{t} \in \mathbb{R}^N \tag{1}$$

Then, for the independent-coding channel and $N = 2$, this becomes:

$$M_{\vec{k}}(\vec{t}) = \langle e^{\vec{t}\cdot\vec{k}} \rangle \tag{2}$$

$$= \sum_{k_1} \sum_{k_2} P(k_1, k_2) e^{t_1 k_1 + t_2 k_2} \tag{3}$$

with

$$P(k_1, k_2) = \int_s \prod_{i=1}^{N} \sum_{\nu_i} P(k_i|\nu_i) P(\nu_i|s) P(s) ds. \tag{4}$$

References

1. Bulmer M. Statistical inference. In: Principles of statistics. Dover Publications; 1979. p. 165–187.
